# Heterogeneity in the efficacy of bosentan for hypertensive nephropathy: a study on individualized benefit prediction models based on pathological subtyping and dynamic trajectories

**DOI:** 10.1080/07853890.2026.2624240

**Published:** 2026-02-06

**Authors:** Xiang Yu, XinYan Gong, BaoLong Wang, YuWei Ji, RiLiGe Wu, WanLing Wang, MengJie Huang, SaSa Nie, GuangYan Cai, XiangMei Chen, Zhe Feng

**Affiliations:** a Department of Nephrology, The First Medical Center of Chinese PLA General Hospital, Chinese PLA Institute of Nephrology, National Key Laboratory of Kidney Diseases, National Clinical Research Center for Kidney Diseases, Beijing Key Laboratory of Kidney Diseases Research, Beijing, China; b Medical Innovation Research Division, Chinese PLA General Hospital, Beijing, China

**Keywords:** Bosentan, endothelin, hypertensive nephropathy, pathological injury, creatinine, treatment response trajectory, precision medicine

## Abstract

**Background:**

Many patients with hypertensive nephropathy progress to chronic kidney disease (CKD). Targeting the core pathophysiological pathway of endothelin-1 activation, this study aimed to elucidate response patterns to the endothelin receptor type A/B (ETA/ETB) antagonist bosentan and construct a personalized prediction model.

**Methods:**

This single-centre retrospective cohort study enrolled 166 patients with hypertension-related nephropathy confirmed by renal biopsy. Risk stratification was performed based on a pathological injury scoring. Three efficacy trajectories were identified using latent variable growth mixture models. Efficacy drivers were screened via random forest algorithms. Finally, early response thresholds were established using receiver operating characteristic (ROC) curves.

**Results:**

Patients in the high-damage group exhibited significantly lower creatinine reduction than those in the low-damage group. Trajectory analysis revealed three patterns: sustained improvement (65.1%), with a 12-month creatinine reduction of 110.8 μmol/L, and deterioration (12.0%), with a deterioration inflection point at 5.3 ± 1.7 months and a subsequent creatinine increase of 40.2 μmol/L. Pathological injury score was the primary determinant of efficacy and was significantly negatively correlated with creatinine reduction (*r* = −0.61). Early response threshold analysis indicated that ΔCr_3M_ ≥ 42.3 μmol/L predicted 12-month efficacy, with an area under the curve (AUC) of 0.79. The early target-achievement group demonstrated significantly greater creatinine reduction than the nonachievement group. Compared with the non-sodium–glucose cotransporter-2 inhibitor (SGLT2i) group, the SGLT2i group demonstrated a 59% reduction in creatinine, while the diabetic subgroup showed a 47.1% smaller reduction than the nondiabetic subgroup.

**Conclusions:**

Bosentan may improve glomerular haemodynamics in patients with hypertensive nephropathy. We propose pathological and early-response thresholds that could guide dynamic, precision interventions before critical inflection points, potentially paving the way for an upgrade from static to proactive management. These findings require validation in larger cohorts.

## 1. Introduction

Hypertensive nephropathy, which is the most severe complication of hypertension for target organs, affects 18.5% of hypertensive patients globally. Approximately, 30% of these patients progress to end-stage renal disease despite treatment with existing RAAS inhibitors [1,2]. A core pathological mechanism involves sustained activation of the endothelin-1 (ET-1) system by intraglomerular hypertension. The binding of ET-1 to endothelin receptor A (ETA) induces vasoconstriction, inflammation, cellular injury and fibrosis, ultimately leading to proteinuria and renal function loss [3]. Bosentan, as a dual nonselective ET receptor antagonist with an affinity ratio of approximately 20:1 for ETA and ETB, theoretically improves renal hemodynamic dysfunction by blocking endothelin receptor type A/B (ETA/ETB) receptors [4,5]. Although renal protective effects have been demonstrated, no ETA-selective receptor antagonists have been approved for treating kidney diseases, and their clinical values remain unclear, particularly in terms of their application to hypertensive nephropathy.

Currently, bosentan is primarily used for treating pulmonary arterial hypertension and paediatric idiopathic pulmonary arterial hypertension [6,7]. Significant evidence gaps exist regarding its efficacy in hypertensive nephropathy, with virtually no clinical research having been conducted. To date, no randomized controlled trials or prospective cohort studies targeting this indication have been reported. Research on endothelin receptor antagonists in nephrology has been focused on diabetic nephropathy [8,9] or chronic kidney disease (CKD) associated with systemic sclerosis (e.g. aldesartan) [10]. However, evidence regarding the efficacy and safety of bosentan for hypertensive nephropathy is entirely absent. Nonselective blockade of ETA/ETB receptors by bosentan may uniquely affect renal haemodynamic regulation; however, the underlying mechanisms remain unclear. Specifically, the influence of renal biopsy pathological severity (global sclerosis rate and vascular lesion grade) on treatment response remains undefined, and the dynamic evolution of therapeutic response lacks systematic investigation. The absence of tools for assessing treatment efficacy heterogeneity severely impedes precision medicine decision-making.

This study is aimed at addressing the challenge of treatment response heterogeneity in bosentan therapy for hypertensive nephropathy. By establishing a pathology-stratified response model, treatment response patterns across patients with varying degrees of renal injury can be revealed. A treatment trajectory evolution map is constructed to identify three characteristic efficacy patterns and their critical temporal milestones. We have developed tools for dynamic efficacy assessment and stratified decision-making, establishing a clinical decision pathway encompassing ‘pathology-based response identification – early efficacy warning – individualized regimen adjustment.’ This study aims to provide an initial exploratory framework for pathologically stratified response patterns and dynamic treatment strategies for bosentan.

## 2. Methods

### 2.1. Study design and population

This single-centre retrospective cohort study included selected patients treated at the First Medical Center of the Chinese PLA General Hospital between 1 January 2020 and 31 May 2024. The inclusion criteria were as follows:

- age ≥ 18 years;
- diagnosis of hypertensive nephropathy according to the Chinese Expert Consensus on Diagnosis and Treatment of Hypertensive Nephropathy (2022);
- complete renal biopsy pathology with reports detailing the global sclerosis rate, vascular lesion grading and interstitial fibrosis stage; receipt of standard dose bosentan (62.5 mg twice daily) therapy for ≥12 months;
- complete follow-up data.

The exclusion criteria, in addition to the inverse conditions of the inclusion criteria, included the following:

- renal biopsy pathology demonstrating any form of glomerulonephritis (including diabetic nephropathy, IgA nephropathy, membranous nephropathy or other primary/secondary glomerular diseases);
- a prior history of kidney transplantation or urinary tract obstruction;
- concurrent secondary causes of hypertension (e.g. renal artery stenosis or pheochromocytoma);
- a baseline eGFR of <15 mL/min/1.73 m^2^.

The bosentan regimen was administered as a standardized clinical protocol at our centre for eligible patients with biopsy-confirmed hypertensive nephropathy, ensuring consistency in the initial therapeutic intervention across the cohort.

The Institutional Review Board of the Chinese PLA General Hospital approved this project and granted informed consent waivers for enrolled patients, as all privacy identifiers were masked or removed (Approval Lun Zi, No. S2025-664). The study strictly adhered to the principles of the Declaration of Helsinki.

### 2.2. Basis for sample size calculation

The study sample size was estimated on the basis of the clinically significant threshold for creatinine change (*δ* = 40 μmol/L) and historical standard deviation (SD = 35.2 μmol/L). When *α* = 0.10 (two-tailed), to enhance sensitivity, a sample size of 160 subjects could be implemented to detect an intergroup difference (80% power).

### 2.3. Case identification

By considering the patient records integrated digital enterprise (PRIDE) platform as the data retrieval source, cases were identified within a specified timeframe on the basis of ICD-10 codes (I12.908 and I12.912) to identify target patients. Patient IDs, admission numbers and hospital identification numbers were exported and saved as Excel spreadsheets. The platform’s medical record browsing function was subsequently used to download and export renal biopsy case reports and medical orders, with cases being identified on the basis of the criteria for natriuresis.

### 2.4. Data collection and variable definitions

Structured data extraction and recording were achieved using the PRIDE platform and its integrated 360° view functionality. These data included demographic characteristics, personal histories, clinical comorbidities, baseline renal function indicators, descriptive outcomes from renal biopsy pathology reports, concomitant medication orders, follow-up metrics and medication safety data. The demographic characteristics included age, sex and body mass index (BMI). The personal history included smoking history, alcohol consumption history, hypertension duration, hypertension classification and peak systolic blood pressure. Clinical comorbidities included diabetes and hyperuricemia. Baseline renal function indicators included the baseline serum creatinine level, baseline estimated glomerular filtration rate (eGFR) and baseline urine protein level, which were the most recent test results prior to the patient’s renal biopsy. Descriptive findings from renal biopsy pathology reports included glomerular volume, global sclerosis rate, arteriolar hyalinosis rate and interstitial fibrosis grade. Concomitant medication orders included angiotensin-converting enzyme inhibitors and sodium–glucose cotransporter 2 inhibitors. Follow-up measurements included serum creatinine levels at four time points (3, 6, 9 and 12 months). Safety data prioritized drug-related adverse events, such as abnormal liver function, frequent headaches, persistent thrombocytopenia, worsening anaemia and sustained hypotension. Concurrently, on the basis of physical examination descriptions in outpatient or inpatient records, the occurrence and progression of peripheral oedema were documented.

### 2.5. Definitions of pathological core variables

The global sclerosis rate in renal biopsy pathology reports could be defined as the percentage of glomeruli with global sclerosis relative to the total number of glomeruli in the biopsy sample. The proportion of glomerular arteriolar hyalinosis could be defined as the percentage of arterioles exhibiting hyalinosis relative to the total number of microscopically visible arterioles. This proportion was converted into a graded scale: <25% was grade 1, 25–50% was grade 2, 50–75% was grade 3 and >75% was grade 4. The degree of renal interstitial fibrosis referred to the extent to which normal interstitial structure was replaced by an abnormal extracellular matrix (collagen I/III). This parameter required the integration of histological features and the Renal Pathology Society (RPS)’s semiquantitative scoring system [11]. Variable conversion was based on severity descriptions: no fibrous ring = grade 0, mild = grade 1, moderate = grade 2 and severe = grade 3.

### 2.6. Development and validation of the pathological injury scoring system

A pathological injury scoring system was developed on the basis of core renal biopsy pathological variables. Data standardization was performed first: the ordinal variable ‘global sclerosis proportion’ underwent *Z* score normalization, while the ordinal categorical variables ‘grade of interstitial fibrosis’ and ‘grade of renal arteriolar hyalinosis’ were coded using dummy variables. Principal component analysis was then performed on the standardized data to extract the composite component explaining the maximum variance. Coefficients for the three pathological indicators were calculated on the basis of the factor loadings from the principal component analysis. Finally, the cases were grouped according to the median pathological injury score and divided into low-injury and high-injury groups.

To assess the validity of the newly developed pathological injury scoring system, we evaluated its correlation with established clinical markers of CKD severity. We hypothesized that a higher pathological score, indicative of more severe structural damage, would correlate with worse baseline kidney function. We computed Spearman’s correlation coefficients between the pathological injury score and two key baseline clinical parameters: eGFR and 24-hour urine protein excretion. Strong and statistically significant correlations in the expected directions would provide evidence for the criterion validity of the scoring system, supporting its utility as a composite indicator of overall kidney damage.

### 2.7. Statistical analysis methods

#### 2.7.1. Descriptive analysis

Multidimensional descriptive statistical methods were employed to systematically compare cohort characteristics and multiple features between the low-damage and high-damage pathological groups. These factors included age, sex, BMI, smoking history, alcohol consumption history, diabetes status, hyperuricemia status, hypertension duration, hypertension classification, peak systolic blood pressure, baseline creatinine level, baseline eGFR and baseline urine protein level. Systematic comparisons validated the clinical rationality of pathological grouping, laying the foundation for subsequent efficacy heterogeneity analysis.

#### 2.7.2. Primary efficacy analysis

Generalized estimating equations (GEEs) were employed to assess the core effect of bosentan treatment [12]. The model used the eGFR as the dependent variable and time points (0/3/6/9/12 months) as core independent variables, adjusting for three confounding factors: age, diabetes status and baseline eGFR. Three key effects were analysed: the main effect of time to assess the overall trend in renal function change, the main effect of grouping to evaluate treatment differences across injury groups, and the time × grouping interaction effect to examine differences in treatment trends across injury groups. The results were presented as regression coefficients (*β*) with 95% confidence intervals, with *p* < 0.05 considered to indicate statistical significance.

#### 2.7.3. Subgroup heterogeneity analysis

Subgroup analyses for treatment heterogeneity were conducted by using GEEs and stratifying according to diabetes status, hyperuricemia status, RAAS blocker use, SGLT2 inhibitor use, age (<60 years or ≥60 years), sex and pathological injury group. After all the models were adjusted for age and baseline eGFR, the time effect coefficient *β* (95% CI) was calculated for each subgroup. The Wald *χ*^2^ test (df = 1) was used to assess the time × subgroup interaction effect, with *p* < 0.05 indicating significant intergroup differences. The final intergroup difference *β* value was defined as the difference between the effect values of the superior subgroup and the reference subgroup.

#### 2.7.4. Dynamic efficacy trajectory analysis

Latent variable growth mixture models (LCMMs) were employed to analyse the dynamic evolution of renal function. The model used serum creatinine levels as the dependent variable and the treatment duration as the independent variable, with three predefined latent trajectory categories: sustained improvement, plateau and deterioration. To account for baseline confounding bias, age and baseline eGFR were included as fixed-effect covariates, whereas diabetes status served as a mixed effect moderator. Model outputs were used to identify distinct efficacy evolution patterns and their critical time points (e.g. plateau onset and deterioration inflection points), with probabilistic density plots visualizing trajectory stratification results.

#### 2.7.5. Exploration of efficacy drivers

An exploratory analysis of efficacy drivers was conducted using a random forest algorithm. First, a candidate variable set was included, and multicollinearity among variables was assessed using variance inflation factors (VIFs), with highly collinear variables (VIFs ≥5) being excluded. After *Z* scores were standardized for continuous variables, a random forest model was constructed with the change in creatinine levels at 12 months after treatment as the response variable. A minimum sample size of 5 per node was set to prevent overfitting. The average increase in the mean squared error (%IncMSE) for each variable was calculated using the permutation importance assessment method. The top five core drivers were selected on the basis of their descending importance scores.

#### 2.7.6. Early response threshold analysis

To establish quantitative standards for early efficacy prediction, the following workflow was employed to determine early response thresholds. First, continuous predictors selected via the random forest algorithm served as candidate indicators. A total reduction in creatinine levels at 12 months reaching the cohort median was defined as an effective response to generate a binary response variable. Receiver operating characteristic (ROC) curve analysis was employed to evaluate the ability of each candidate indicator to predict an effective response at 12 months. The indicator with the optimal predictive efficacy (area under the curve, AUC value) and its threshold were selected for clinical decision-making. The optimal threshold was determined using the Youden index maximization principle, dividing patients into early-response achievers and nonachievers. Diagnostic metrics such as sensitivity and specificity were then calculated for this threshold.

#### 2.7.7. Propensity score matching analysis

To address potential confounding and isolate the independent effect of the pathological injury score, we performed a propensity score matching (PSM) analysis. Patients in the high-injury and low-injury groups were matched 1:1 without replacement using the nearest neighbour method, with a calliper width set to 0.02. The matching was based on the key covariates: age, sex and baseline eGFR. The balance of covariates between the matched groups was assessed using standardized mean differences (SMDs), with an SMD < 0.10 indicating good balance.

**Figure 1.**
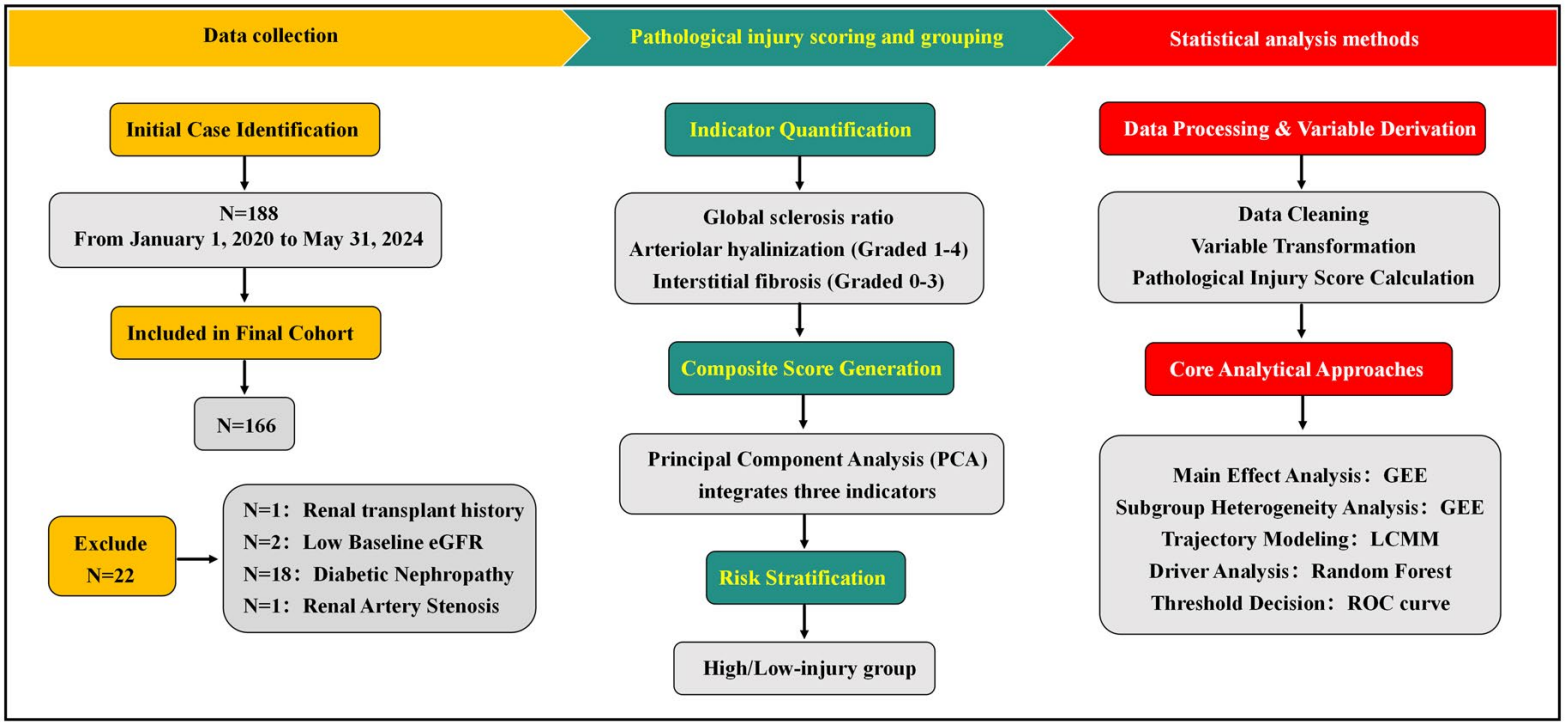
Study flowchart.

All the retrieved data and derived variables were uniformly stored in CSV files. The complete data processing workflow is detailed in Figure 1. Data reading and modelling were performed using R-Studio statistical software (Version 4.1.0, R Foundation for Statistical Computing, Vienna, Austria), with subsequent operations being conducted via custom coding that associated each variable with its timestamp and type. All the *p* values were two-tailed, and values less than 0.05 were considered to indicate statistical significance.

## 3. Results

In this study, 188 patients from the First Medical Center of the PLA General Hospital who underwent renal biopsy and who received a pathological diagnosis of hypertensive nephropathy were screened. Among these patients, 22 were excluded because they met the exclusion criteria, resulting in a final cohort of 166 patients. All 166 included patients received the standardized initial dose of bosentan (62.5 mg twice daily), confirming adherence to the treatment protocol. Patients were divided into high-damage and low-damage groups on the basis of the median pathological damage score (7.2 points). The high-damage group was older (*p* < 0.05), had a higher incidence of diabetes and hyperuricemia (*p* < 0.05), had a longer duration of hypertension (*p* < 0.05), had greater hypertension severity (*p* < 0.05), had higher baseline serum creatinine and urine protein levels, and had a lower baseline eGFR (*p* < 0.05) than the low-damage group (Table 1).

**Table 1.** Baseline characteristics and pathological stratification of hypertensive nephropathy patients.

Characteristic	Overall (*n* = 166)	Low-injury group (*n* = 83)	High-injury group (*n* = 83)	*p* Value
Demographic characteristics				
Age (years)	57.8 (49.0–64.5)	53.2 (47.5–59.5)	62.3 (57.5–68.5)	0.001
Male	107 (64.5)	52 (62.7)	55 (66.3)	0.601
BMI (kg/m^2^)	24.9 (22.9–27.3)	24.4 (22.6–26.4)	25.1 (23.1–27.8)	0.385
Personal history				
Smoking history	68 (41)	33 (39.8)	35 (42.2)	0.752
Alcohol history	51 (30.7)	25 (30.1)	26 (31.3)	0.869
Hypertension duration (years)	10.3 (7.0–14.2)	8.2 (5.2–10.3)	12.3 (9.1–16.5)	<0.001
Hypertension grade				0.008
Grade 1	28 (16.9)	19 (22.9)	9 (10.8)	
Grade 2	78 (47)	42 (50.6)	36 (43.4)	
Grade 3	60 (36.1)	22 (26.5)	38 (45.8)	
Peak systolic pressure (mmHg)	170 (159–180)	166 (157–175)	174 (164–184)	0.208
Clinical comorbidities				
Diabetes	48 (28.9)	12 (14.5)	36 (43.4)	<0.001
Hyperuricemia	59 (35.5)	18 (21.7)	41 (49.4)	<0.001
Baseline renal function indicators				
Serum creatinine (μmol/L)	175.0 (99.5–360.2)	150.5 (120.8–195.3)	240.8 (185.6–385.5)	<0.001
eGFR (ml/min/1.73 m^2^)	35.0 (23.0–79.8)	48.5 (30.2–75.6)	28.7 (15.3–62.4)	<0.001
24-Hour urine protein (g/24 h)	1.25 (0.72–2.42)	0.80 (0.43–1.55)	1.95 (0.95–3.25)	<0.001
Concomitant medications				
RAASi	92 (55.4)	48 (57.8)	44 (53)	0.542
SGLT2i	74 (44.6)	35 (42.2)	39 (47)	0.532

BMI: body mass index; eGFR: estimated glomerular filtration rate; RAASi: renin–angiotensin aldosterone system inhibitors; SGLT2i: sodium glucose cotransporter 2 inhibitor.

Data are presented as median (IQR) or frequency (%).

During the 12-month follow-up period, five (3.01%) out of 166 patients experienced transient elevations in liver enzymes. Among these patients, three temporarily discontinued medication use and recovered after 2 weeks; however, no patients permanently discontinued medication usage. Two patients (1.20%) had a history of neurology outpatient visits due to headache symptoms. Seven patients (4.22%) developed new oedema symptoms, and four patients (2.41%) experienced worsening of preexisting oedema. No other drug-related adverse events, such as persistent thrombocytopenia, worsening anaemia or persistent hypotension, were recorded.

### 3.1. Primary efficacy analysis

The primary efficacy analysis demonstrated that bosentan treatment significantly improved renal function in patients with hypertensive nephropathy (*p* < 0.001). Compared with baseline creatinine levels, serum creatinine levels decreased significantly at 3, 9 and 12 months of treatment (*p* < 0.001) and plateaued at 6 months (*p* = 0.076). Patients in the high pathological injury group had a baseline serum creatinine concentration that was 90.3 μmol/L higher than that in the low injury group (*p* < 0.001). Interaction analysis revealed that the treatment benefit appeared to be significantly diminished in the high injury group compared with that in the low injury group, with an actual decrease in serum creatinine concentration of 9.8 μmol/L per 3-month period (Table 2).

**Table 2.** Time effects and pathological stratification response patterns to bosentan therapy.

Variable	*β* (95% CI)	Std. err.	Wald *χ*^2^	*p* Value
Time main effect				
3 months vs. baseline	−33.5 (−39.2 to −27.8)	2.91	132.5	<0.001
6 months vs. baseline	−32.0 (−37.8 to −26.2)	2.95	117.6	0.076
9 months vs. baseline	−67.8 (−74.3 to −61.3)	3.32	167.9	<0.001
12 months vs. baseline	−92.6 (−99.8 to −85.4)	3.68	233.7	<0.001
Group main effect				
Baseline sCr difference	+90.3 (62.5–118.1)	14.18	40.6	<0.001
Interaction effect				
Time × high-injury group	+9.8 (2.1–17.5)	3.93	6.2	0.013

GEE models are adjusted for age, baseline eGFR and diabetes; *β* values are presented in μmol/L; the time main effect indicates a serum creatinine change from baseline at each time point (negative values = decline); the group main effect reflects a baseline creatinine difference between high- and low-injury groups (positive values = higher baseline in high-injury group); and the interaction effect quantifies reductions in creatinine decline per 3 months in high- vs. low-injury group (positive values = attenuated treatment response).

### 3.2. Efficacy analysis in the propensity score-matched cohort

To address the concern that the treatment response difference might be attributed to baseline imbalances in age, sex and renal function, we performed a PSM analysis. After matching, 58 well-balanced pairs (*n* = 116) were generated. The matched cohorts showed no significant differences in age, sex or baseline eGFR (all SMD < 0.10; see Supplementary Table S1). Critically, in this matched cohort where these key confounders were balanced, the significant difference in the treatment response between the low-injury and high-injury groups persisted. The reduction in serum creatinine per 3 months remained significantly greater in the low-injury group (29.5 μmol/L) compared to the high-injury group (16.8 μmol/L), with a mean difference of 12.7 μmol/L (95% CI: 2.8–22.6; *p* = 0.012).

### 3.3. Validation of the pathological injury scoring system

The pathological injury score demonstrated strong and statistically significant correlations with established clinical markers of kidney disease severity, supporting its criterion validity. There was a significant negative correlation between the pathological score and baseline eGFR (Spearman’s *r* = −0.67, *p* < 0.001), indicating that higher pathological damage was associated with poorer kidney function. Conversely, a significant positive correlation was observed between the pathological score and baseline 24-hour urine protein (Spearman’s *r* = 0.58, *p* < 0.001), confirming that greater structural damage was associated with increased protein leakage.

### 3.4. Subgroup analysis

Subgroup analysis revealed significant heterogeneity in bosentan treatment efficacy. Pathological injury severity, diabetes status and SGLT2 inhibitor use significantly modulated the bosentan response. Patients in the low pathological injury group achieved a 30.8 μmol/L reduction in serum creatinine concentration every 3 months, representing a 94% increase compared with that in the high pathological injury group (*p* = 0.003). Nondiabetic patients had a 25.8 μmol/L decrease, which was 91% greater than that of diabetic patients (*p* = 0.038). SGLT2 inhibitor users had a 24.8 μmol/L decrease, which was 59% greater than that of nonusers (15.6 μmol/L) (*p* = 0.011). No significant differences in efficacy were observed in the hyperuricemia subgroup, RAAS inhibitor-treated subgroup, sex subgroup or age subgroup (*p* > 0.05) (Table 3).

**Table 3.** Subgroup analysis of heterogeneity in serum creatinine response to bosentan.

Subgroup	*N*	Time effect *β* (95% CI)	*p* Value	Intergroup difference *β*	Intergroup *p* value
Diabetes					
No	118	−25.8 (−31.2 to −20.4)	<0.001	Reference	
Yes	48	−13.5 (−20.3 to −6.7)	<0.001	+12.3	0.038
Hyperuricemia					
No	107	−24.5 (−29.6 to −19.4)	<0.001	Reference	
Yes	59	−15.0 (−21.2 to −8.8)	<0.001	+9.5	0.088
RAASi					
No	74	−21.3 (−26.8 to −15.8)	<0.001	Reference	–
Yes	92	−18.1 (−23.2 to −13.0)	<0.001	+3.2	0.407
SGLT2i					
No	92	−15.6 (−20.8 to −10.4)	<0.001	Reference	–
Yes	74	−24.8 (−30.2 to −19.4)	<0.001	+9.2	0.011
Age, years					
<60	99	−22.4 (−27.3 to −17.5)	<0.001	Reference	–
≥60	67	−17.8 (−23.6 to −12.0)	<0.001	+4.6	0.291
Sex					
Male	107	−21.0 (−26.5, −15.5)	<0.001	Reference	−
Female	59	−24.5 (−30.8, −18.2)	<0.001	+3.5	0.350
Pathological group					
Low-injury group	83	−30.8 (−35.6 to −26.0)	<0.001	Reference	–
High-injury group	83	−15.9 (−21.8 to −10.0)	<0.001	+14.9	0.003

ΔCr: creatinine change per 3 months; RAASi: renin–angiotensin aldosterone system inhibitors; SGLT2i: sodium glucose cotransporter 2 inhibitor.

GEE models are uniformly adjusted for age and baseline eGFR; the group difference is *β* (effect value of the advantaged subgroup minus the reference subgroup).

### 3.5. Dynamic efficacy trajectory analysis

**Figure 2.**
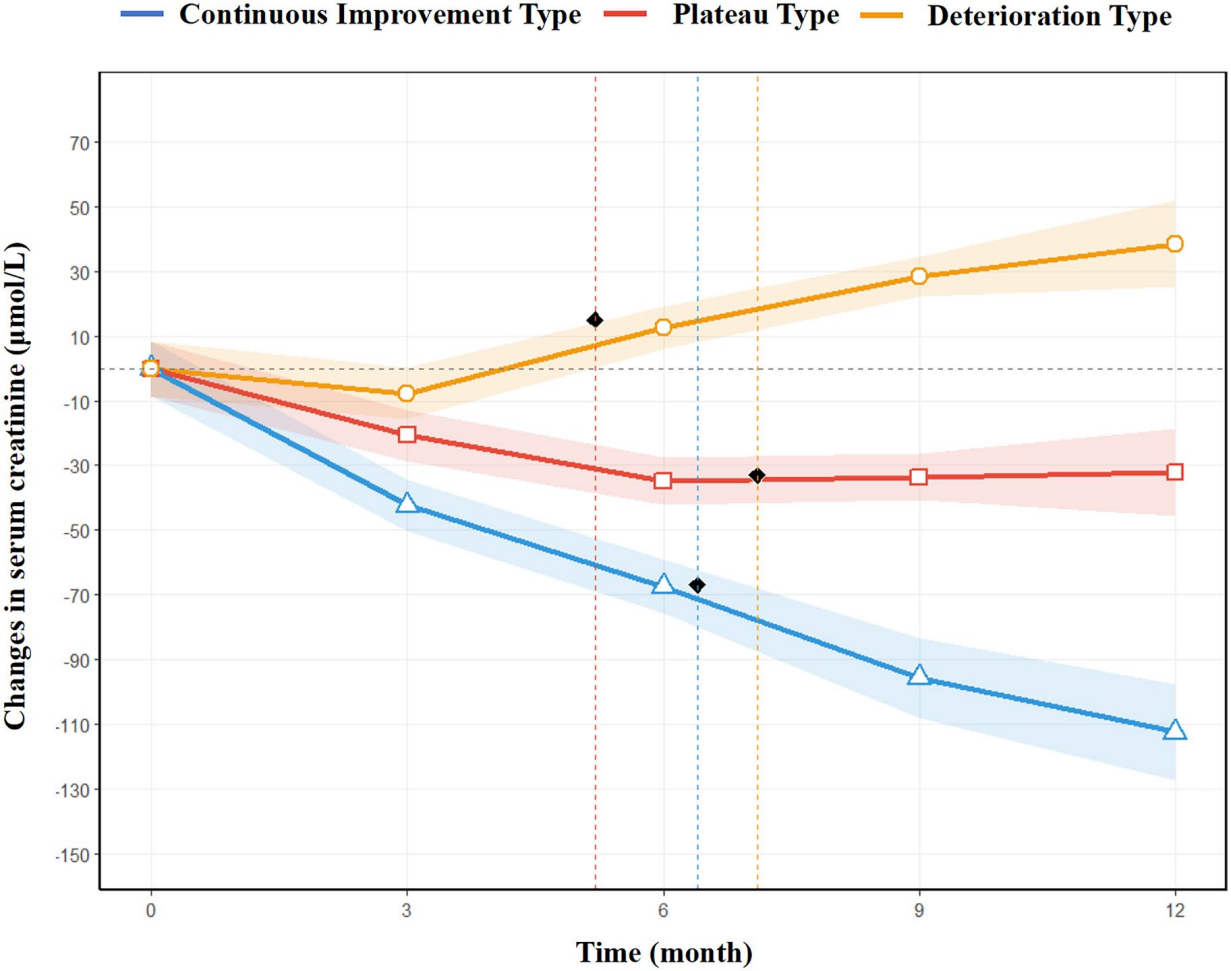
Trajectories of serum creatinine changes over time. *Note*: This line chart displays three distinct renal response trajectories identified by latent class mixed models: continuous improvement (blue), plateau (red) and deterioration (yellow). The *Y*-axis represents the change in serum creatinine (μmol/L), and the *X*-axis is time (months). The shaded areas represent the 95% confidence intervals. The continuous improvement group shows a sustained decline, the plateau group stabilizes after initial improvement, and the deterioration group exhibits an upward trend after the inflection point at 5.3 months.

Dynamic efficacy trajectory analysis successfully revealed three patterns of therapeutic evolution: sustained improvement (65.1%), plateau (22.9%) and deterioration (12.0%). Patients in the continuous improvement pattern demonstrated significant renal function enhancement, with a cumulative 12-month creatinine reduction of 110.8 μmol/L and an acceleration inflection point at 6.3 ± 1.1 months. Patients in the plateau pattern entered a stagnation phase after 6 months, with a 12-month creatinine reduction of 83.5 μmol/L and a stagnation onset at 7.0 ± 1.4 months. Patients in the deterioration pattern exhibited a deterioration inflection point at 5.3 ± 1.7 months, with a 12-month increase in creatinine concentration of 40.2 μmol/L. Baseline characteristics differed significantly among the three patterns. Compared with patients with the sustained improvement pattern, patients with the deterioration pattern were older, had higher pathological injury scores, had a higher proportion of severe pathological injury, and had a higher incidence of diabetes. Early treatment response characteristics were particularly critical. Patients with sustained improvement achieved a 41.5 μmol/L decrease in creatinine concentration at 3 months, whereas patients with deterioration showed only a 7.5 μmol/L reduction, indicating a highly significant difference (*p* < 0.001) (Table 4 and Figure 2).

**Table 4.** Clinical characteristics and key parameters of three efficacy trajectory subtypes.

Characteristic/indicator	Sustained improvement (*n* = 108)	Plateau (*n* = 38)	Deterioration (*n* = 20)	*p* Value
Trajectory characteristics				
12-Month sCr change (μmol/L)	−110.8 (−124.6 to −97.0)	−83.5 (−95.2 to −71.8)	40.2 (25.6–54.8)	<0.001
Key transition point (months)	6.3 ± 1.1	7.0 ± 1.4	5.3 ± 1.7	
Baseline characteristics				
Age (years)	54.0 (47.5–59.5)	61.5 (56.0–66.5)	65.5 (62.0–70.5)	<0.001
Pathological injury score	4.7 (3.9–5.5)	7.3 (6.5–8.1)	8.3 (7.7–8.9)	<0.001
High-injury group, *n* (%)	27 (25)	34 (89.5)	20 (100)	<0.001
Early response marker				
3-Month sCr reduction (μmol/L)	−41.5 (−48.3 to −34.7)	−18.8 (−25.6 to −12.0)	−7.5 (−14.3 to −0.7)	<0.001
Diabetes, *n* (%)	15 (13.9)	18 (47.4)	15 (75)	<0.001
SGLT2i, *n* (%)	42 (38.9)	20 (52.6)	4 (20)	0.032

Recent class mixed models are adjusted for age and baseline eGFR; diabetes status is modelled as a mixture effect; *β* is the monthly change in creatinine; transition point is defined as the time when the slope is ≥0.

### 3.6. Exploration of efficacy drivers

**Figure 3.**
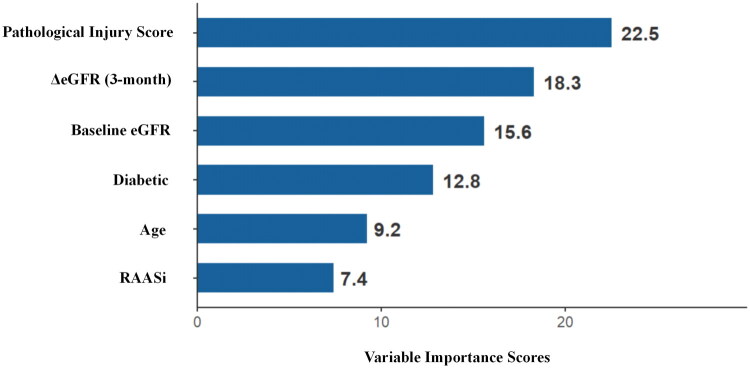
Variable importance scores of predictors. *Note*: This bar chart illustrates the importance scores of predictors in the random forest model for renal function outcomes. The pathological injury score was the most influential variable. Higher scores indicate a greater contribution to the model’s predictive accuracy.

Random forest analysis revealed the following key drivers of treatment response ranked by importance: pathological injury score, ΔCr_3M_, baseline eGFR, diabetes status and age. Compared with the other factors, the pathological injury score was significantly increased (*p* < 0.001) and was significantly negatively correlated with the 12-month decrease in creatinine (*r* = −0.61; *p* < 0.001) (Figure 3).

### 3.7. Validation of the early response threshold

**Figure 4.**
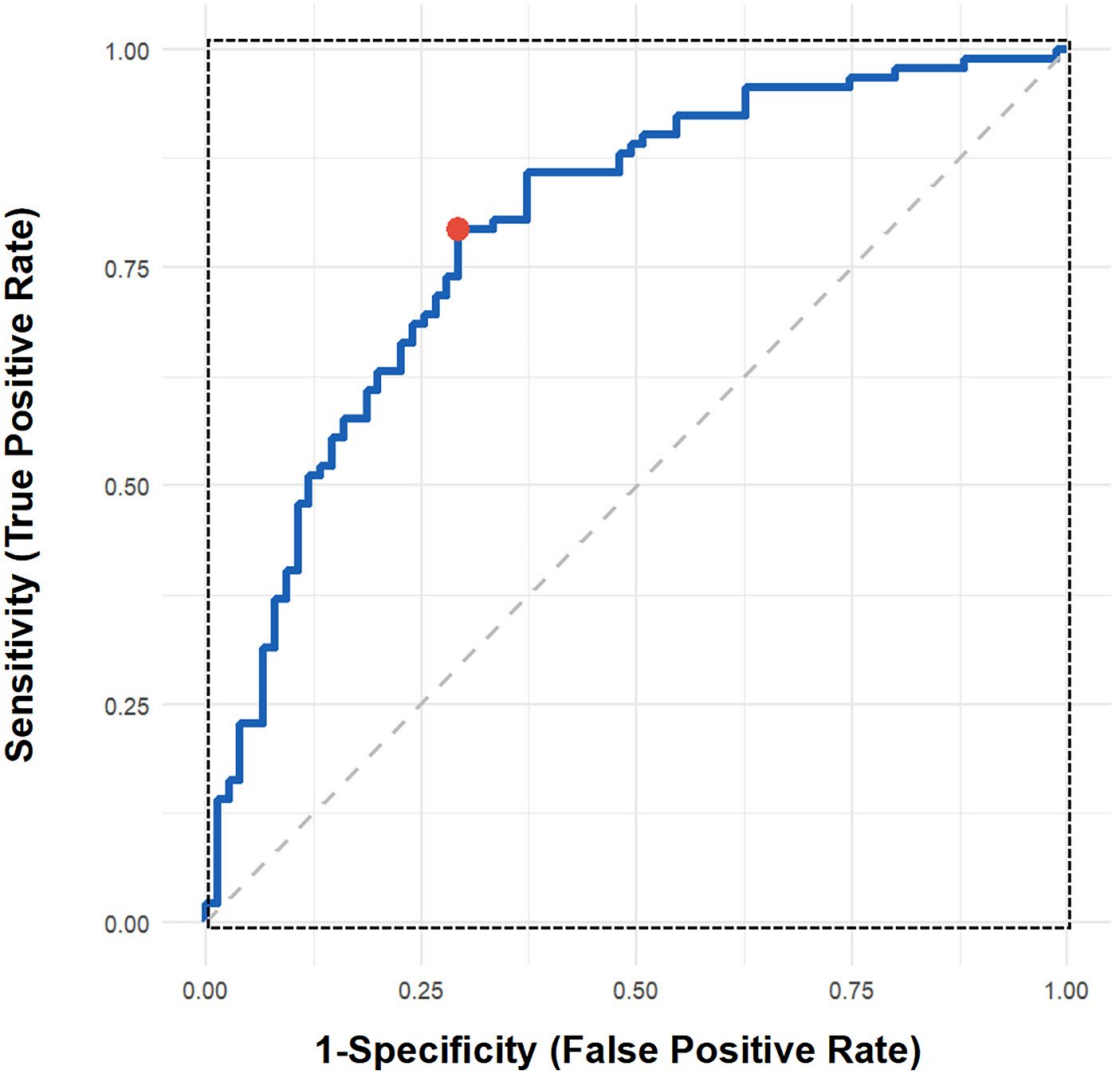
Receiver operating characteristic (ROC) curve. *Note*: The ROC curve evaluates the predictive performance of ΔCr_3M_ (3-month creatinine change) for the 12-month renal response.

**Figure 5.**
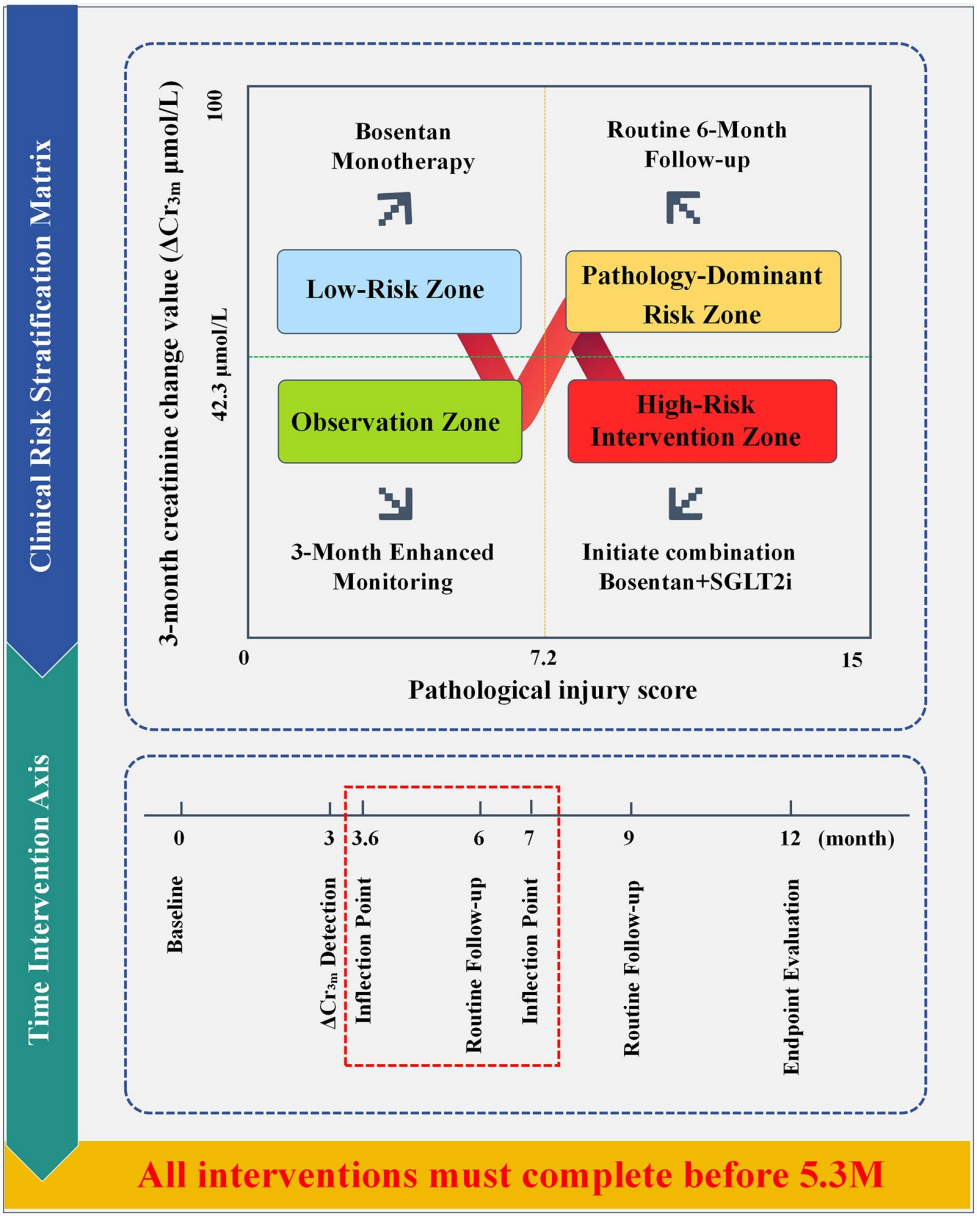
Clinical risk stratification matrix and intervention timeline. *Note*: This integrated figure consists of a risk stratification matrix based on the pathological injury score (*X*-axis) and ΔCr_3M_ at 3 months (*Y*-axis), coupled with an intervention timeline below. The matrix is divided into four zones: low-risk (blue, bosentan monotherapy), observation (green, enhanced monitoring), pathology-dominant risk (yellow, consider additional diagnostics) and high-risk intervention (red, initiate combination therapy with bosentan and SGLT2i). The timeline indicates critical time points for assessment and intervention. All interventions must be completed before the key window period ending at 5.3 months.

ROC curve analysis established the predictive value of ΔCr_3M_ for a 12-month efficacy, with an AUC of 0.79 (Figure 4). Youden’s index maximization was used to determine the cutoff value at 42.3 μmol/L, yielding a sensitivity of 82.4% and a specificity of 73.6%. Grouping by this threshold revealed that 92 patients were in the early target group (ΔCr_3M_ ≥42.3 μmol/L). Their 12-month creatinine reduction (105.6 ± 31.2 μmol/L) significantly exceeded that of the nonachievement group (29.8 ± 37.4 μmol/L) (*p* < 0.001), with a positive predictive value of 84.8%. This threshold was highly correlated with the dynamic trajectory classification, as 85.0% of deteriorating patients had a ΔCr_3M_ of <42.3 μmol/L (Figure 5).

## 4. Discussion

To our knowledge, this study is among the first to suggest distinct pathological response patterns to bosentan treatment in hypertensive nephropathy through a dynamic trajectory modelling system, filling an evidence gap for endothelin receptor antagonists in this field. These findings indicate that the severity of renal biopsy pathological damage is the primary determinant of treatment heterogeneity. Compared with the low-damage group, the high-damage group showed a 48.4% reduction in the 3-month creatinine decrease rate. Moreover, the random forest algorithm confirms the pathological injury score as the dominant core driver of the therapeutic response. Patients who achieved a serum creatinine reduction exceeding 42.3 μmol/L at 3 months demonstrate a 3.5-fold increase in improvement rates at 12 months. Notably, 85.0% of patients with worsening disease exhibit insufficient response characteristics early in treatment.

ET may participate in the development and progression of kidney disease through multiple mechanisms. ETA is highly expressed in podocytes, where it binds to ET1 with high affinity, inducing podocyte injury [13,14]. Similarly, mesangial cells can directly produce ET-1, which binds to ET receptors via autocrine activity, leading to mesangial cell contraction, proliferation and increased mesangial matrix [5]. Additionally, animal studies have confirmed that elevated ET-1 expression increases renal fibrosis in mice [15]. Consequently, ERAs hold promise for renal protection by improving glomerular haemodynamics through ETA-mediated vasodilation, reducing glomerular perfusion pressure and mitigating mesangial matrix accumulation, inflammation and fibrosis, ultimately decreasing glomerular permeability and proteinuria. Given the distinct mechanisms underlying ETA and ETB, current research is focused on selective ERAs to avoid ETB-mediated ET1 clearance effects. Atrasentan and sitaxsentan, as ETA receptor antagonists, have yielded positive outcomes in animal studies [16,17]. However, scholars have confirmed that ambrisentan has similar renoprotective effects in an ischemia–reperfusion rat model, with both agents reducing renal apoptosis, tissue injury and inflammatory responses [18]. Additionally, in cyclosporine-induced nephrotoxic hypertensive rats, while both macitentan and bosentan reduce blood pressure and improve haemodynamic changes, only bosentan can reverse cyclosporine-induced nephrotoxicity. Thus, bosentan appears more adept at reversing hemodynamic alterations than other factors [19].

This study reveals a decrease in the therapeutic efficacy of bosentan in the high pathological injury group. These findings suggest that this phenomenon may be deeply linked to the pathophysiological mechanisms of the ET-1 system. Recent research has confirmed that under pathological conditions such as diabetes or hypertension, elevated glucose levels, acidosis, and the presence of insulin, angiotensin II and proinflammatory cytokines can increase ET-1 concentrations. This increase induces vasoconstriction via ETA receptors and mesangial cell proliferation, potentially causing harmful effects such as glomerular hyperfiltration or podocyte injury. This process accelerates the global sclerotic process, ultimately leading to proteinuria and a reduced GFR [20–23]. Although bosentan blocks ETA/ETB receptors [7,24], the extent of hemodynamic improvement is significantly limited in patients with extensive glomerulosclerosis and severe interstitial fibrosis due to microvascular structural damage. This phenomenon may explain why the high-damage group achieves only 52% of the 3-month creatinine reduction observed in the low-damage group.

Our pathological score addresses a distinct clinical need compared to prognostic tools like the kidney failure risk equation (KFRE). While KFRE predicts long-term renal survival [25]. Our score aims to stratify the short-term, therapy-specific hemodynamic response to bosentan based on underlying renal structure. Its predictive value, which persisted after PSM for age and eGFR, confirms that it provides complementary information not captured by these functional parameters alone. Future direct comparisons with KFRE are warranted.

Notably, these findings contrast sharply with those of studies of ET antagonists in diabetic nephropathy. In the latter context, elevated circulating ET-1 levels in diabetic patients have been reported in animal models and short-term proof-of-concept studies, in which highly selective ET-A receptor antagonists reduce proteinuria and improve glomerulosclerosis [24,26]. However, the results of the present study demonstrate that bosentan remains ineffective in the high-damage subgroup without diabetes. Notably, SGLT2 inhibitor use significantly increases bosentan efficacy in this study, potentially because SGLT2 inhibitors improve glomerular hyperfiltration, reduce inflammation and directly inhibit ET-1 synthesis [27–29]. Previous studies have confirmed that the combination of aldesartan and losartan improves podocyte numbers and structures while reducing proteinuria in BTBR ob/ob mice, mirroring human outcomes [26,30]. Although the efficacy heterogeneity of RAAS blockers has not been demonstrated in subgroup analyses in this study, the interaction effects of their combination warrant further investigation.

Subgroup analysis has revealed the effects of metabolic factors on bosentan efficacy: diabetic status reduces creatinine levels by 47.1%, which is directly correlated with hyperglycaemia-induced ET-1 synthesis [20]. While hyperuricemia is correlated with baseline injury, it has no independent effect, suggesting that uric acid may indirectly weaken the treatment response by accelerating structural damage [31,32]. Notably, no significant synergistic effect can be observed with RAAS blockers, likely because of overlapping effects from their shared influence on glomerular haemodynamics. Furthermore, the synergistic interaction between SGLT2 inhibitors and bosentan offers novel therapeutic insights, particularly for patients with diabetic nephropathy complicated by hypertension, where combination therapy may improve outcomes through multitargeted intervention. These findings further underscore the necessity of combined pathological–metabolic stratification, particularly for patients with diabetes and high pathological damage. The active exploration of alternative, more effective and mechanistically complementary treatment strategies, such as the combination of bosentan and SGLT2 inhibitors, is warranted. Furthermore, the exploratory analysis by sex, informed by the biology of differential endothelin receptor density, did not reveal a statistically significant difference in treatment response [33,34]. This lack of association may be attributable to the sample size, and the observed numerical trends warrant confirmation in larger, dedicated studies.

Identifying efficacy trajectories provides a dynamic perspective for evaluating treatment outcomes in hypertensive nephropathy patients. A key finding is the predictive value of early creatinine changes for deterioration trajectories. Among patients with a decrease in serum creatinine concentration of <42.3 μmol/L at 3 months after treatment, 85.0% (17/20) progress to a deteriorating level. These findings reaffirm previous findings highlighting the predictive role of early subtle renal function changes in treatment outcomes [35]. This study further reveals that if patients on a deteriorating trajectory do not receive intensive intervention before the inflection point of deterioration (95% CI: 3.6–7.0 months) is reached, their rate of renal function deterioration will significantly accelerate. This critical time window may closely align with the pathological process in which the glomerular filtration pressure surpasses the compensatory threshold. These findings suggest that identifying high-risk patients (e.g. those whose ΔCr_3M_ is <42.3 μmol/L) before the glomerular transmembrane pressure reaches irreversible injury thresholds creates an optimal window for intensive intervention. This early warning–intervention dynamic management model upgrades the annual static assessment recommended by KDIGO guidelines into a precise, proactive decision-making approach, offering a novel strategy for overcoming treatment bottlenecks in hypertensive nephropathy [36].

A potential innovative aspect of this study is the proposal of a dual-dimensional decision framework of pathological injury-early response. On the basis of random forest analysis, a pathological injury score >7.2 points has been identified as the core driver, whereas early creatinine dynamics (ΔCr_3M_ <42.3 μmol/L) has ranked second in importance after pathological score and baseline eGFR. On this basis, a dual-trigger clinical pathway is proposed. Specifically, for patients meeting both high-risk features, immediate initiation of bosentan + sodium–glucose cotransporter-2 inhibitor (SGLT2i) combination therapy with intensified intervention before the deterioration inflection point (95% CI: 3.6–7.0 months) can reduce ESRD risk. This finding could potentially contribute to evolving the traditional static pathological stratification model to a dynamic monitoring model. Mechanistically, early inadequate response may reflect either a sudden decrease in residual renal unit ET receptor density or irreversible vascular remodelling [5], which is significantly correlated with histologically confirmed extensive glomerulosclerosis and moderate-to-severe interstitial fibrosis. The early threshold strategy advances the clinical intervention window from the 6–12 month assessment recommended by KDIGO guidelines to 3 months [36]. Initiating regimen intensification before the deterioration inflection point holds promise for reducing ESRD risk.

This study has certain limitations. First, as a single-centre retrospective cohort study, the core validity of the pathological injury score identified via random forest methods and the robustness of the early response threshold across broad populations have yet to be confirmed. Second, the pathological injury scoring system, while demonstrating strong internal validity, is a novel tool that relies on renal biopsy histological interpretation, the level of standardization in this interpretation impacts stratification accuracy, and the score itself requires external validation in future independent cohorts. Third, the simplified dichotomous classification of diabetes status overlooks continuous effects such as glucose control gradients. Fourth, systematic analysis of proteinuria dynamics during treatment was limited by data availability. Finally, evidence-based pathways for alternative treatment regimens in high-risk groups remain unestablished, necessitating the establishment of further RCT studies to clarify second-line treatment options.

## 5. Conclusions

This exploratory research describes the pathophysiological response patterns and proposes personalized treatment decision pathways for bosentan therapy in patients with hypertensive nephropathy. Dynamic trajectory modelling identified three characteristic efficacy evolution patterns, suggesting that renal biopsy pathological damage severity is a key determinant of treatment heterogeneity; a significantly reduced response was observed in patients with high levels of damage. This study provides initial evidence that early renal function changes after bosentan therapy have predictive value for long-term outcomes. Furthermore, a quantitative response threshold based on the reduction in creatinine levels at 3 months of treatment was defined. These findings provide a rationale for considering intervention during the therapeutic window. The proposed dual-lock clinical pathway, based on pathological injury scores and early response thresholds, could enable precise risk stratification and early warning of treatment response, suggesting a potential strategy for overcoming the bottleneck in personalized treatment for hypertensive nephropathy. However, these findings and the proposed model are hypothesis-generating and require validation in larger, prospective studies before clinical application.

## Supplementary Material

Supplementary Table S1.docx

## Data Availability

The data that support the findings of this study are available from the Medical Innovation Research Division, Chinese PLA General Hospital, but there are restrictions regarding the availability of these data, which were used under license for the present study and are not publicly available. However, the data are available from the corresponding author (Zhe Feng) upon reasonable request and with the permission of the Medical Innovation Research Division.

## References

[1] Johansen KL, Chertow GM, Gilbertson DT, et al. US Renal Data System 2022 Annual Data Report: epidemiology of kidney disease in the United States. Am J Kidney Dis. 2023;81(3 Suppl. 1):A8–A11. doi: 10.1053/j.ajkd.2022.12.001. 36822739 PMC10807034

[2] Yang C, Gao B, Wang F, et al. Executive summary for the China Kidney Disease Network (CK-NET) 2017–2018 Annual Data Report. Kidney Int. 2025;107(6):980–984. doi: 10.1016/j.kint.2024.12.017. 40404261

[3] Wang C, Wang Z, Zhang W. The potential role of complement alternative pathway activation in hypertensive renal damage. Exp Biol Med. 2022;247(9):797–804. doi: 10.1177/15353702221091986. PMC9134763 35473318

[4] Bearzi P, Navarini L, Currado D, et al. Bosentan effect on echocardiographic systolic pulmonary arterial pressure in systemic sclerosis-related pulmonary hypertension: a systematic review and metaanalysis. Clin Exp Rheumatol. 2024;42(8):1615–1622. doi: 10.55563/clinexprheumatol/xbdtb5. 38819960

[5] Martínez-Díaz I, Martos N, Llorens-Cebrià C, et al. Endothelin receptor antagonists in kidney disease. Int J Mol Sci. 2023;24(4):3427. doi: 10.3390/ijms24043427. 36834836 PMC9965540

[6] Rivera-Lebron BN, Risbano MG. Ambrisentan: a review of its use in pulmonary arterial hypertension. Ther Adv Respir Dis. 2017;11(6):233–244. doi: 10.1177/1753465817696040. 28425346 PMC5933647

[7] Enevoldsen FC, Sahana J, Wehland M, et al. Endothelin receptor antagonists: status quo and future perspectives for targeted therapy. J Clin Med. 2020;9(3):824. doi: 10.3390/jcm9030824. 32197449 PMC7141375

[8] Yu S, Sun J, Guo X. Efficacy and adverse effects of atrasentan in patients with diabetic nephropathy: a meta-analysis. Altern Ther Health Med. 2024;30(1):454–459. 37820676

[9] Empitu MA, Rinastiti P, Kadariswantiningsih IN. Targeting endothelin signaling in podocyte injury and diabetic nephropathy-diabetic kidney disease. J Nephrol. 2025;38(1):49–60. doi: 10.1007/s40620-024-02072-w. 39302622

[10] Stern EP, Host LV, Wanjiku I, et al. Zibotentan in systemic sclerosis-associated chronic kidney disease: a phase II randomised placebo-controlled trial. Arthritis Res Ther. 2022;24(1):130. doi: 10.1186/s13075-022-02818-6. 35650639 PMC9158153

[11] Nivedita J, Gunabooshanam B, Balasubramanian S. Clinicopathological analysis and its correlation with various classes of lupus nephritis. Cureus. 2025;17:e78065. 40013193 10.7759/cureus.78065 PMC11864095

[12] Ito T, Sugasawa S. Grouped generalized estimating equations for longitudinal data analysis. Biometrics. 2023;79(3):1868–1879. doi: 10.7759/cureus.78065. 35819419

[13] Kohan DE, Bedard Patricia W, Jenkinson C, et al. Mechanism of protective actions of sparsentan in the kidney: lessons from studies in models of chronic kidney disease. Clin Sci. 2024;138(11):645–662. doi: 10.1042/CS20240249. PMC11139641 38808486

[14] Lassén E, Daehn IS. Clues to glomerular cell chatter in focal segmental glomerulosclerosis: via endothelin-1/ETR. Kidney Int Rep. 2021;6(7):1758–1760. doi: 10.1016/j.ekir.2021.05.013. 34307972 PMC8258585

[15] Benigni A, Buelli S, Kohan DE. Endothelin-targeted new treatments for proteinuric and inflammatory glomerular diseases: focus on the added value to anti-renin–angiotensin system inhibition. Pediatr Nephrol. 2021;36(4):763–775. doi: 10.1007/s00467-020-04518-2. 32185491

[16] Bądzyńska B, Vaneckova I, Sadowski J, et al. Effects of systemic and renal intramedullary endothelin-1 receptor blockade on tissue NO and intrarenal hemodynamics in normotensive and hypertensive rats. Eur J Pharmacol. 2021;910:174445. doi: 10.1016/j.ejphar.2021.174445. 34492284

[17] Colafella KMM, Neves KB, Montezano AC, et al. Selective ETA vs. dual ETA/B receptor blockade for the prevention of sunitinib-induced hypertension and albuminuria in WKY rats. Cardiovasc Res. 2020;116:1779–1790. doi: 10.1093/cvr/cvz260. 31593221

[18] Kazimoglu H, Uysal E, Dokur M, et al. Comparison of the protective effects of selective endothelin-a receptor antagonist, ambrisentan, and dual endothelin-A/B receptor antagonist, bosentan, in experimental renal ischemia reperfusion injury. Bratisl Lek Listy. 2020;121(8):547–553. doi: 10.4149/BLL_2020_091. 32726116

[19] Caires A, Fernandes GS, Leme AM, et al. Endothelin-1 receptor antagonists protect the kidney against the nephrotoxicity induced by cyclosporine-A in normotensive and hypertensive rats. Braz J Med Biol Res. 2017;51(2):e6373. doi: 10.1590/1414-431X20176373. 29267497 PMC5731326

[20] Raina R, Chauvin A, Chakraborty R, et al. The role of endothelin and endothelin antagonists in chronic kidney disease. Kidney Dis. 2020;6(1):22–34. doi: 10.1159/000504623. PMC6995952 32021871

[21] Sindhu D, Sharma GS, Kumbala D. Management of diabetic kidney disease: where do we stand? A narrative review. Medicine. 2023;102(13):e33366. doi: 10.1097/MD.0000000000033366. 37000108 PMC10063294

[22] Yang Y, Xu G. Update on pathogenesis of glomerular hyperfiltration in early diabetic kidney disease. Front Endocrinol. 2022;13:872918. doi: 10.3389/fendo.2022.872918. PMC9161673 35663316

[23] Helal I, Fick-Brosnahan GM, Reed-Gitomer B, et al. Glomerular hyperfiltration: definitions, mechanisms and clinical implications. Nat Rev Nephrol. 2012;8(5):293–300. doi: 10.1038/nrneph.2012.19. 22349487

[24] Zhou Y, Chi J, Huang Y, et al. Efficacy and safety of endothelin receptor antagonists in type 2 diabetic kidney disease: a systematic review and meta-analysis of randomized controlled trials. Diabet Med. 2021;38(1):e14411. doi: 10.1111/dme.14411. 33000477

[25] Tangri N, Stevens LA, Griffith J, et al. A predictive model for progression of chronic kidney disease to kidney failure. JAMA. 2011;305(15):1553–1559. doi: 10.1001/jama.2011.451. 21482743

[26] Heerspink HJL, Parving HH, Andress DL, et al. Atrasentan and renal events in patients with type 2 diabetes and chronic kidney disease (SONAR): a double-blind, randomised, placebo-controlled trial. Lancet. 2019;393(10184):1937–1947. doi: 10.1016/S0140-6736(19)30772-X. 30995972

[27] Clemmer JS, Yen TE, Obi Y. Modeling the renoprotective mechanisms of SGLT2 inhibition in hypertensive chronic kidney disease. Physiol Rep. 2023;11(21):e15836. doi: 10.14814/phy2.15836. 37957121 PMC10643202

[28] Wang M, Zuo L. Cardiorenal benefits of SGLT2 inhibitors in patients with chronic kidney disease and concomitant hypertension. Cardiorenal Med. 2025;15(1):496–509. doi: 10.1159/000545622. 40258345

[29] Yang S, Zhao L, Mi Y, et al. Effects of sodium–glucose cotransporter-2 inhibitors and aldosterone antagonists, in addition to renin–angiotensin system antagonists, on major adverse kidney outcomes in patients with type 2 diabetes and chronic kidney disease: a systematic review and network meta-analysis. Diabetes Obes Metab. 2022;24(11):2159–2168. doi: 10.1111/dom.14801. 35712807

[30] Hudkins KL, Wietecha TA, Steegh F, et al. Beneficial effect on podocyte number in experimental diabetic nephropathy resulting from combined atrasentan and RAAS inhibition therapy. Am J Physiol Renal Physiol. 2020;318(5):F1295–F1305. doi: 10.1152/ajprenal.00498.2019. 32249614

[31] Watanabe K, Nakayama M, Yamamoto T, et al. Different clinical impact of hyperuricemia according to etiologies of chronic kidney disease: Gonryo study. PLOS One. 2021;16(3):e0249240. doi: 10.1371/journal.pone.0249240. 33765101 PMC7993817

[32] Hung YH, Huang CC, Lin LY, et al. Uric acid and impairment of renal function in non-diabetic hypertensive patients. Front Med. 2021;8:746886. doi: 10.3389/fmed.2021.746886. PMC8818871 35141237

[33] Kittikulsuth W, Sullivan JC, Pollock DM. ET-1 actions in the kidney: evidence for sex differences. Br J Pharmacol. 2013;168(2):318–326. doi: 10.1111/j.1476-5381.2012.01922.x. 22372527 PMC3572558

[34] Stanhewicz AE, Wenner MM, Stachenfeld NS. Sex differences in endothelial function important to vascular health and overall cardiovascular disease risk across the lifespan. Am J Physiol Heart Circ Physiol. 2018;315(6):H1569–H1588. doi: 10.1152/ajpheart.00396.2018. 30216121 PMC6734083

[35] Li Q, Li G, Li D, et al. Early and minimal changes in serum creatinine can predict prognosis in elderly patients receiving invasive mechanical ventilation: a retrospective observational study. J Intensive Med. 2024;4(3):368–375. doi: 10.1016/j.jointm.2023.10.003. 39035610 PMC11258507

[36] Kidney Disease: Improving Global Outcomes (KDIGO) CKD Work Group. KDIGO 2024 clinical practice guideline for the evaluation and management of chronic kidney disease. Kidney Int. 2024;105(4S):S117–S314. doi: 10.1053/j.ajkd.2024.08.003. 38490803

