# Supplementary material for: Heterogeneity in the efficacy of bosentan for hypertensive nephropathy: a study on individualized benefit prediction models based on pathological subtyping and dynamic trajectories
Source: Ann Med. 2026 Feb 6;58(1):2624240. doi: 10.1080/07853890.2026.2624240 (PMC12885016; doi:10.1080/07853890.2026.2624240)
Supplement: Supplementary Table S1.docx [file IANN_A_2624240_SM0182.docx]

| **Variable** | **Low-Injury Group**  **(n=58)** | **High-Injury Group (n=58)** | **P Value** |
| --- | --- | --- | --- |
| Age (years), median (IQR) | 61.0 (55.5, 66.5) | 62.3 (57.5, 68.5) | 0.535 |
| Male, n (%) | 37 (63.8%) | 36 (62.1%) | 0.845 |
| Baseline eGFR (ml/min/1.73 m²), median (IQR) | 29.8 (18.1, 58.0) | 28.7 (15.3, 62.4) | 0.901 |
| ΔSerum Creatinine per 3 months (μmol/L), mean (95% CI) | -29.5 (-36.2, -22.8) | -16.8 (-23.5, -10.1) | 0.012 |

**Supplementary Table S1. Propensity Score Matching: Baseline and Outcome Analysis**

Note: Propensity score matching was performed 1:1 on age, sex, and baseline eGFR. P values are from Wilcoxon rank-sum tests (continuous) or Chi-square tests (categorical), except for the treatment response (Δ serum creatinine), which is from a GEE model in the matched cohort.
